# An Inverse Method to Estimate the Root Water Uptake Source-Sink Term in Soil Water Transport Equation under the Effect of Superabsorbent Polymer

**DOI:** 10.1371/journal.pone.0159936

**Published:** 2016-08-09

**Authors:** Renkuan Liao, Peiling Yang, Wenyong Wu, Shumei Ren

**Affiliations:** 1 State Key Laboratory of Simulation and Regulation of Water Cycles in River Basins, China Institute of Water Resources and Hydropower Research, Beijing, China; 2 College of Water Conservancy and Civil Engineering, China Agricultural University, Beijing, China; Estacion Experimental del Zaidin, SPAIN

## Abstract

The widespread use of superabsorbent polymers (SAPs) in arid regions improves the efficiency of local land and water use. However, SAPs’ repeated absorption and release of water has periodic and unstable effects on both soil’s physical and chemical properties and on the growth of plant roots, which complicates modeling of water movement in SAP-treated soils. In this paper, we proposea model of soil water movement for SAP-treated soils. The residence time of SAP in the soil and the duration of the experiment were considered as the same parameter *t*. This simplifies previously proposed models in which the residence time of SAP in the soil and the experiment’s duration were considered as two independent parameters. Numerical testing was carried out on the inverse method of estimating the source/sink term of root water uptake in the model of soil water movement under the effect of SAP. The test results show that time interval, hydraulic parameters, test error, and instrument precision had a significant influence on the stability of the inverse method, while time step, layering of soil, and boundary conditions had relatively smaller effects. A comprehensive analysis of the method’s stability, calculation, and accuracy suggests that the proposed inverse method applies if the following conditions are satisfied: the time interval is between 5 d and 17 d; the time step is between 1000 and 10000; the test error is ≥ 0.9; the instrument precision is ≤ 0.03; and the rate of soil surface evaporation is ≤ 0.6 mm/d.

## Introduction

The study on law of soil water movement has great significance in understanding the process of material transport and energy transfer in Soil-Plant-Atmosphere Continuum (SPAC) system, and water uptake by crop roots is one of the most important processes in the transport and energy transfer of the soil in the farmland. To understand the dynamics of soil water uptake by roots is very important for the rational development of irrigation system, the improvement of crop water use efficiency and the guarantee of stable and high yield of crops. In general, it is difficult for us to obtain the dynamics of soil water movement, so modeling the dynamic process of water uptake by plant roots and water movement in soils has become an important and common research method in the field of irrigation and rain-fed agriculture. So far, most existing soil water movement models are using the Richards equation to describe the dynamics of water transport in soil[1–4], and models that take into consideration root water uptake have been constructed by adding a source/sink term of root water uptake (rate of water uptake) to the Richards equation[5–7]. However, the rate of root water uptake is not measurable under the current technical condition. To address this, a method of obtaining a reliable “measurement” for root water uptake has been used widely in prior research on water and nutrient uptake by plant roots. This method measures water uptake using inverse methods of estimating the water uptake source/sink term added to the Richards equation[8–10], which makes it possibility to study the relationship between soil moisture and crop water uptake. This method, proposed by Zuo Q, Shi JC et al[11,12], is based on two continuous soil moisture profiles to calculate the root water uptake rate, and has been successfully applied in research into water uptake by wheat’s roots. Superabsorbent polymers (SAPs) are a type of water-absorbing polymer, which can be made of starch, chitosan, acrylic, lignin, etc[13–17], widely used in construction field, medical and health, environmental protection and water saving agriculture. For example, in the field of construction, SAP can enhance the sustainability of concrete and minimize disposal in ready mixed concrete operation[18]; In medical and health, SAP can be used as drug delivery or flocculant[19]; In environmental protection, SAP was selected as a moderate water shutoff agent for water production control[20]. More commonly, SAP was used as a water-saving agent in rain-fed agriculture[21]. When absorption and swelling, the three-dimensional hydrogel network of SAP is wrapped in a membrane structure to hold the water. The water is retained because of osmotic pressure and molecular forces, and the three-dimensional network unfolds continuously until it reaches equilibrium and stops absorbing water[22]. Studies have shown that when a SAP is applied to soil around plant roots, the SAP rapidly absorbs the water in the soil, which reduces deep seepage loss, and then water is gradually released to the plants. This process improves the efficiency of soil water use[23–25]. However, some studies have suggested that the repeated water absorption and release mechanism of SAPs also exerts periodic and unstable influences on the pattern of soil water movement by affecting the soil’s physical and chemical properties, local microbial communities, and root growth. This adds to the inherent difficulty and complexity of modeling water movement in SAP-treated soils[26–29]. Han YG et al [30,31] proposed an analytical solution by establishing a one-dimensional model of soil water movement under the effect of SAP, however, this model considers the water movement time and the residence time of SAP in the soil as two independent variables and does not provide a solution to the rate of root water uptake. This creates limitations for in-depth research into water management systems using SAP-treated soils.

In this paper, we propose a soil water movement model based on the Richards equation, and we equate the time of water movement with the residence time of SAP, both designated as *t*, and takes into account root water uptake. We then use an inverse iteration method to derive a numerical solution for the rate of root water uptake in order to provide guidance for further research on modeling root water uptake in soil under the effect of SAP.

## Materials and Methods

### Model of Soil Water Movement under Normal Conditions

When considering plant growth and evaporation from the soil surface, measuring one-dimensional vertical movement of water in unsaturated soil is usually described using the Richards equation (Eq 1):

$$\left\{ \begin{array}{l} \frac{\partial \theta }{\partial t}=\frac{\partial }{\partial z}\left[D\left(\theta \right)\frac{\partial \theta }{\partial z}\right]-\frac{\partial K\left(\theta \right)}{\partial z}-S\left(z,t\right)\\ \theta \left(z,0\right)=\theta_{0}\left(z\right)0\leq z\leq Lr\\ \theta \left(Lr,t\right)=\theta_{1}\left(t\right)t>0\\ -D\left(\theta \right)\frac{\partial \theta }{\partial z}+K\left(\theta \right)=-E\left(t\right)t>0 \end{array}\right.$$ Eq 1

where *D(θ)* is the unsaturated diffusivity of soil, *K(θ)* is the unsaturated hydraulic conductivity of soil, *S(z*,*t)* is the source/sink term of root water uptake, and *E(t)* is the rate of soil surface evaporation.

### Model of Soil Water Movement under the Effect of SAP

Since the SAP’s water retention ability varies over time, the unsaturated diffusivity and unsaturated hydraulic conductivity of SAP-treated soil are functions of both the soil moisture content *θ* and time *t* rather than only the soil moisture content *θ*. The time-varying functions are denoted by *D(θ*,*t)* and *K(θ*,*t)*. When root water uptake and soil surface evaporation are considered, measuring the one-dimensional vertical movement of water in unsaturated soil under the effect of SAP is described using the equation below:

$$\left\{ \begin{array}{l} \frac{\partial \theta }{\partial t}=\frac{\partial }{\partial z}\left[D\left(\theta ,t\right)\frac{\partial \theta }{\partial z}\right]-\frac{\partial K\left(\theta ,t\right)}{\partial z}-S\left(z,t\right)\\ \theta \left(z,0\right)=\theta_{0}\left(z\right)0\leq z\leq Lr\\ \theta \left(Lr,t\right)=\theta_{1}\left(t\right)t>0\\ -D\left(\theta ,t\right)\frac{\partial \theta }{\partial z}+K\left(\theta ,t\right)=-E\left(t\right)t>0 \end{array}\right.$$ Eq 2

where *D(θ*,*t)* and *K(θ*,*t)* respectively denote time-varying functions for unsaturated diffusivity and unsaturated hydraulic conductivity; the other parameters are the same as in Eq 1.

### Solving the Model of Soil Water Movement under the Effect of SAP

Typically, when analytically solving the equation by means of the Laplace transform and integration by parts, the unsaturated diffusivity *D(θ*,*t)* in the Richards equation is first replaced by the average diffusivity *D*. This is because the equation in Eq 2 is a nonlinear equation. However, because the pattern of variation in this hydraulic parameter is an important indicator of the effect of the SAPs, averaging it to simplify the problem can greatly reduce the significance of the proposed model of soil water movement under the effect of SAP. The implicit differentiation method[32] was used in this study in order to ensure the model had correct physical significance and to make the equation easier to solve. This was done because of the great difficulty in analytically solving the Richards equation with time-varying hydraulic parameters. The implicit differentiation is unconditionally convergent when used to solve equations and it did not limit the ranges of values in the determination of the time step and distance step as discussed in the subsequent section describing numerical testing.

Additionally, the modeling of one-dimensional vertical movement of water in unsaturated soil is based on the following basic assumptions:

1. SAP is applied to a rigid soil whose properties vary over time and the effect from its volumetric change is negligible.
2. The soil’s hydraulic parameters don’t change during measurement.

### Solving the Source/Sink Term of Root Water Uptake in the Model of Soil Water Movement under the Effect of SAP

The inverse iteration method developed by Zuo et al[11,12]. was used to solve the source/sink term of root water uptake. The two continuous profiles of measured soil moisture are designated as *θ(z*, *0)* and *θ(z*, *T)*. The decrease in soil moisture, resulting from root water uptake over the period from *0* to *T* at different depths, is represented by *Δθ(z*, *0~T)*, in which *T* is the time interval between two successive measurements of root water uptake. The average rate of root water uptake over the period between 0 and T can be expressed as *S(z*,*0~T) = Δθ(z*,*0~T)/T*.

First, let *S = 0* and implicitly differentiate the water movement model including time-varying hydraulic parameters (Eq 5–2) based on initial measurements of soil moisture (*t = 0*) to obtain the value of *θ*^*(1)*^*(z*_*i*_,*T)* when *t = T* in the first iteration. This first iteration ignores the influence of *S* by letting *S = 0*. Calculate *Δθ*^*(1)*^*(z*_*i*_,*0~T)* by subtracting *θ*^*(T)*^*(z*_*i*_,*T)* from *θ*^*(1)*^*(z*_*i*_,*T)* and dividing the resulting value by *T*. The result of this division is then input into the original equation as an approximation of the average rate of water uptake *S*^*(1)*^, which is defined by *S*^*(1)*^ *= Δθ*^*(1)*^*/T*, to obtain *θ*^*(2)*^*(z*_*i*_,*T)* in the second iteration. The influence of *S* is considered in the second and subsequent iterations by letting *S ≠ 0*. If the value of *Δθ*^*(2)*^*(z*_*i*_,*0~T)* is smaller than the error threshold (the mean square relative error), the iterative process is terminated; otherwise, calculate *S*^*(2)*^ using the relationship *S*^*(2)*^ *= S*^*(1)*^*+Δθ*^*(2)*^*/T* and input the result into the original equation for another iteration. This process should be repeated until the aforementioned termination criterion is met.

The R language was used to program the inverse method of calculating the source/sink term of root water uptake.

### Plotting and Error Test

Analysis and plotting were performed in *Scientific Data Analysis and Visualization (SciDAVis*1.D8). The differences between the measured values and the values predicted by the model were assessed using the root mean square error (RMSE, a measure of dispersion of estimated values), maximum absolute error (MAE, a measure of maximum deviation of estimated values from measured values), and overall relative error (ORE, a measure of overall deviation of estimated values from measured values).

$$\begin{array}{l} RMSE={\left[\frac{1}{n}\sum_{}^{}{\left(S_{\left(measured\right)}-S_{\left(estimated\right)}\right)}^{2}\right]}^{1/2}\\ MAE=\underset{i=1,2,3\ldots n}{Max}\left|S_{\left(measured\right)}-S_{\left(estimated\right)}\right|\\ ORE=\left|\frac{\sum_{}^{}S_{\left(measured\right)}-\sum_{}^{}S_{\left(estimated\right)}}{\sum_{}^{}S_{\left(measured\right)}}\right|\times 100\% \end{array}$$

where *S*_*measured*_ represents a measured value and *S*_*estimated*_ represents an estimated value.

### Stability Analysis of the Inverse Method

In this study we used the theoretical root water uptake model developed by Shao AJ et al. [33,34] to investigate the relationship between transpiration and root water uptake. This model is represented by the following equation:

$$S=ET\times A\times \left[e^{-B{\left(\ln Z-C\right)}^{2}}\right]/Z$$ Eq 3

where *ET* represents the rate of evapotranspiration (mm/d); *Z* is the relative depth and is defined by *Z* = *z*/*L*_*r*_(*t*); *A*, *B* and *C* are empirical coefficients, with *A* being a one-dimensional coefficient expressed in mm, and *B* and *C* are non-dimensional. *L*_*r*_(*t*) is the depth of the bottom of the zone that supplies water to plant roots and can be expressed as:

$$L_{r}\left(t\right)=150\left(1-e^{-1.380813T}\right)$$ Eq 4

where *T* represents relative time and is defined by *T* = *t*/*M*, in which *M* is the duration of corn growth (*d*), and *t* is the length of time since the corn seeds were sown into the soil.

The soil’s physical parameters and root parameters presented in previous studies[33,34] are described here. The soil in the test field is divided into two layers: the upper 70 cm-thick layer consists of silty soil and the lower layer consists of sandy loam. The unsaturated hydraulic conductivity and soil-water-characteristic curve of the silty soil can be expressed as *D* = 0.00148*e*^15.99^ and *θ* = 0.48*e*^-0.00143|*h*|^ (0 ≤ |*h*| ≤ 129.2), respectively. The unsaturated hydraulic conductivity of the sandy loam can be described by *D* = 54.9*θ*^3.614^ and its soil-water-characteristic curve can be described by *θ* = 0.50*e*^-0.00146|*h*|^ (0 ≤ |*h*| ≤ 168.64) or *θ* = 13.893|*h*|^-0.719^ (|*h*| > 168.64). The expressions of unsaturated diffusivity of the two soil typeswere derived from the existing soil-water-characteristic curves in previous studies and the relationship *θ* = *θ*_r_ + (*θ*_s_—*θ*_r_)/[1 + (*αh*)^n^]^m^. The parameters in the theoretical water uptake model are as follows: *A* = 8.600827 × 10^−4^–1.18926 × 10^−3^(*T*-0.591)^2^, *B* = 1.662603, *C* = -1.30806; *M* = 88 d, *ET* = 3 mm/d, and the error threshold for terminating the iterative process is 1×10^−5^. The test of the stability of the inverse method described above considers the following factors: time interval and time step, the soil’s hydraulic parameters, errors in the measured values of soil moisture, instrument precision, layering of the soil, and boundary conditions. Table 1 shows the values of these parameters. The numerical testing included the following steps:

1. The moisture movement parameters and the parameters in the theoretical root water uptake model were input, and the inverse method’s control conditions (e.g. time step, space step, instrument precision, and boundary conditions) were determined.
2. The initial soil moisture profile *θ*(*z*, 0) and theoretical root water uptake *S*(*z*, 0) were input, and the equation describing soil water movement (Eq 2) was solved using implicit differentiation to obtain the theoretical moisture distribution in the soil profile at *t*, denoted as *θ*(*z*, *t*). Since errors might occur when measuring soil moisture, a vector error (VE) with a normal distribution was used as a disturbance term (indicated by *per* value in the program) in order to make the measured values more accurate. This resulted in the equation: θ*(*z*,*t*) = *θ*(*z*,*t*) + VE.
3. The algebraic equation (Eq 5) was used to fit the values of θ*(*z*, *t*) obtained in Step (2), yielding a continuous and smooth distribution curve of θ**(*z*, *t*):
  $$\theta^{**}\left(z,t\right)=r_{1}+r_{2}\left(z-Z\right)+r_{3}{\left(z-Z\right)}^{2}+\ldots +r_{n}{\left(z-Z\right)}^{n-1}$$ Eq 5
  where *r*_1_, *r*_2_, …, *r*_n_ are fitting parameters and *Z* is the cumulative average of *z*_1_, *z*_2_, …, *z*_n_. The fitting process started from *n* = 3 and continued until the MAE of the calculated values was smaller than the threshold value for instrument precision *w* for error control.
4. The inverse method described in Section 5.3 was used to infer the average water uptake over the period from *t*_1_ (initial time) to *t*_2_ (ending time), denoted as *S*_*estimated*_(*z*, *t*_1_-*t*_2_), from the distribution curve of θ*(*z*, *t*) obtained by Step (3).
5. The average errors between the theoretical and calculated values of the root water uptake were analyzed using RMSE, MAE, and ORM, in order to examine the accuracy of the simulation.

**Table 1:** Selection of parameters for numerical testing.

Numerical testing		—————————————————Parameter———————————————						
		Soil type	*E*	*D*	*K*	*Θ*(*z*,0)	*per*	*w*
1	Time interval	Sandy loam	0.3	0.00148*e*^15.99*θ*^	2.0733*θ*^7.9663^	0.3	1	0.01
	Time step	Sandy loam	0.3	0.00148*e*^15.99*θ*^	2.0733*θ*^7.9663^	0.3	1	0.01
2	*D*	Sandy loam	0.3	Experimental value	2.0733*θ*^7.9663^	0.3	1	0.01
	*K*	Sandy loam	0.3	0.00148*e*^15.99*θ*^	Experimental value	0.3	1	0.01
3	Test error	Sandy loam	0.3	0.00148*e*^15.99*θ*^	2.0733*θ*^7.9663^	0.3	Experimental value	0.01
	Instrument precision	Sandy loam	0.3	0.00148*e*^15.99*θ*^	2.0733*θ*^7.9663^	0.3	1	Experimental value
4	Layered soil	Experimental value	0.3	Experimental value	Experimental value	0.3	1	0.01
	Soil surface evaporation	Sandy loam	Experimental value	0.00148*e*^15.99*θ*^	2.0733*θ*^7.9663^	0.3	1	0.01

**Fig 1:**
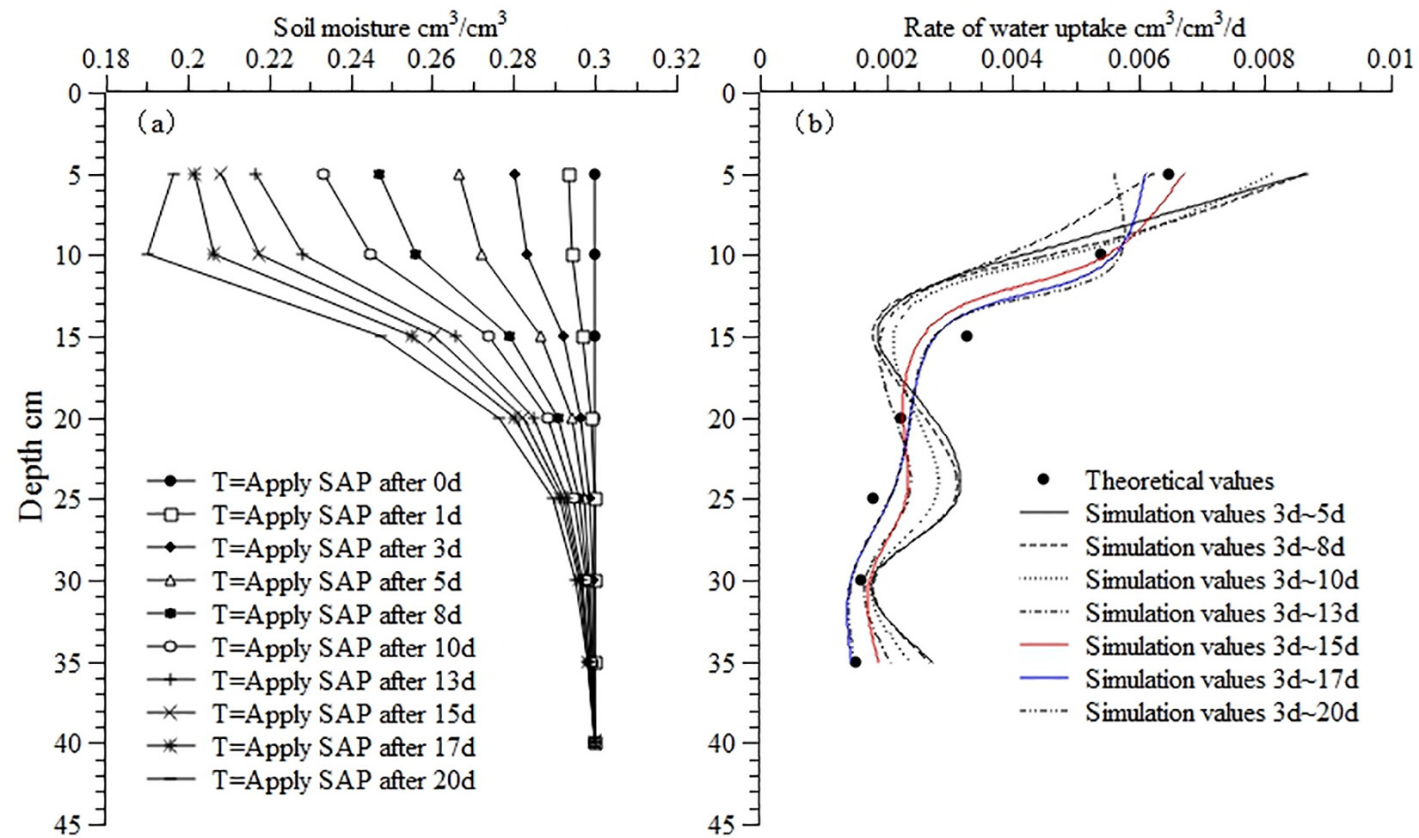
Theoretical moisture and calculated water uptake distribution in the soil profile at different times. (a) Theoretical moisture distribution; (b) Calculated water uptake distribution. Use inverse method to calculate distribution of moisture (apply SAP after 0, 1, 3, 5, 8, 10, 13, 15, 17, 20d) and water uptake (from apply SAP 3d to 5d, 3d to 8d, 3d to 10d, 3d to 13d, 3d to 15d, 3d to 17d, 3d to 20d) in the soil profile at different times. Theoretical values are calculated from the root water uptake model developed by Shao AJ et al [33,34] (*S* = *ET*×*A*×[*e^-B*(*lnZ-C*)^2^]/Z).

Table 1 shows the parameters of four numerical tests including main influencing factors: time interval, time step, D, K, test error, instrument precision, layered soil and soil surface evaporation. The “*E*” stands for evaporation, The “*D*” stands for unsaturated diffusivity, The “*K*” stands for unsaturated hydraulic conductivity, The “*Θ*(*z*,0)” stands for distribution of initial moisture in soil profile, The “*per*” stands for test errors, The “*w*” stands for instrument precisions, The inverse method is used to calculate distribution of moisture and water uptake in soil profile by testing experimental value (from Fig 1 to Fig 8).

## Results

### (1) Influence of time interval and time step

Fig 1 shows the theoretical soil moisture distribution and the corresponding water uptake distribution at different times. It is assumed that the initial soil moisture is normally distributed. Theoretical values of soil moisture in the soil profile at different times (Fig 1A) were calculated using the water movement equation (Eq 1) and the theoretical water uptake model (Eq 3). When calculating root water uptake at different time intervals the theoretical value of soil moisture at 3 d was used as the initial value, because the practical distribution of soil moisture is not uniform. Then, the distribution of soil moisture at different time intervals was calculated using the inverse method, as shown in Fig 1B. The figure shows that as the time interval increased from 2 d to 22 d the degree of correlation between the calculated and theoretical values of root water uptake first increased and then decreased. The results of the error analysis (Table 2) show that the differences between the theoretical and calculated values reached the lowest levels when the time interval was between 12 d and 14 d, and the corresponding ORE fell in the range of 3.53% to 5.83%. As the time interval increased from 2 d to 10 d, the RMSE fell between 5.87×10^−4^ and 8.42×10^−4^, the MAE fell between 5.18×10^−4^ and 2.18×10^−3^, and the ORE, fell between 6.52 and 12.68%. Based on these results, the theoretical soil moisture distribution at a time interval of 12 d (i.e. the soil moisture distribution from 3 d to 15 d) was chosen for further analysis at different time steps.

**Table 2:** Error analysis between theoretical and calculated values.

Numerical testing	Factor	Level	RMSE	MAE	ORE (%)
Test 1	Time interval	2 d	1.83×10^−3^	3.07×10^−3^	22.97
		5 d	1.74×10^−3^	3.02×10^−3^	23.95
		7 d	1.32×10^−3^	2.32×10^−3^	18.54
		10 d	1.15×10^−3^	2.07×10^−3^	16.05
		12 d	5.31×10^−4^	9.88×10^−4^	5.83
		14 d	3.95×10^−4^	6.97×10^−4^	3.53
		17 d	5.87×10^−4^	1.21×10^−3^	6.52
		22 d	8.42×10^−4^	2.18×10^−3^	12.68
	Time step	10	5.76×10^−4^	1.07×10^−3^	5.31
		100	5.16×10^−4^	9.88×10^−4^	3.83
		1000	4.51×10^−4^	7.12×10^−4^	1.50
		10000	4.59×10^−4^	7.50×10^−4^	2.63
Test 2	Unsaturated hydraulic conductivity	100 *D*	1.64×10^−3^	3.26×10^−3^	27.75
		10 *D*	5.85×10^−4^	1.27×10^−3^	3.01
		5 *D*	5.35×10^−4^	9.57×10^−4^	3.17
		0.5 *D*	1.28×10^−3^	3.00×10^−3^	19.95
		0.1 *D*	1.14×10^−2^	2.95×10^−2^	120.11
	Unsaturated diffusivity	100 *K*	1.87×10^−3^	4.24×10^−3^	28.54
		10 *K*	5.52×10^−4^	9.95×10^−4^	5.13
		5 *K*	4.47×10^−4^	8.20×10^−4^	2.79
		0.5 *K*	4.23×10^−4^	7.06×10^−4^	3.21
		0.1 *K*	4.23×10^−4^	7.05×10^−4^	3.18
		0.01 *K*	4.23×10^−4^	7.05×10^−4^	3.16
		0.001 *K*	5.97×10^−4^	9.47×10^−4^	5.81
Test 3	Test error	*per* = 0.95	5.15×10^−4^	1.15×10^−3^	0.28
		*per* = 0.9	6.51×10^−4^	1.49×10^−3^	0.55
		*per* = 0.85	1.48×10^−3^	2.56×10^−3^	21.04
		*per* = 0.8	1.75×10^−3^	3.03×10^−3^	25.64
		*per* = 0.7	2.37×10^−3^	4.12×10^−3^	38.03
	Instrument precision	*w* = 0.01	4.27×10^−4^	7.12×10^−4^	3.41
		*w* = 0.03	7.84×10^−4^	1.68×10^−3^	7.14
		*w* = 0.05	2.06×10^−3^	4.05×10^−3^	23.13
		*w* = 0.1	4.76×10^−3^	1.01×10^−2^	44.66
Test 4	Layered soil	sandy loam+silty soil	5.86×10^−4^	1.02×10^−3^	5.92
	Soil surface evaporation	*E* = 0.03	4.90×10^−4^	8.52×10^−4^	4.45
		*E* = 0.1	7.41×10^−4^	1.41×10^−3^	7.33
		*E* = 0.3	4.27×10^−4^	7.12×10^−4^	3.45
		*E* = 0.6	9.68×10^−4^	1.50×10^−3^	5.59

Table 2 shows the error analysis between theoretical and calculated values including main influencing factors: time interval, time step, *D*, *K*, test error, instrument precision, layered soil and soil surface evaporation. The “*E*” stands for evaporation, The “*D*” stands for unsaturated diffusivity, The “*K*” stands for unsaturated hydraulic conductivity, The “*per*” stands for test errors, The “*w*” stands for instrument precisions. The “*RMSE*” stands for the root mean square error, which is a measure of dispersion of estimated values. The “*MAE*” stands for maximum absolute error, which is a measure of maximum deviation of estimated values from measured values. The “*ORE*” stands for overall relative error, which is a measure of overall deviation of estimated values from measured values.

**Fig 2:**
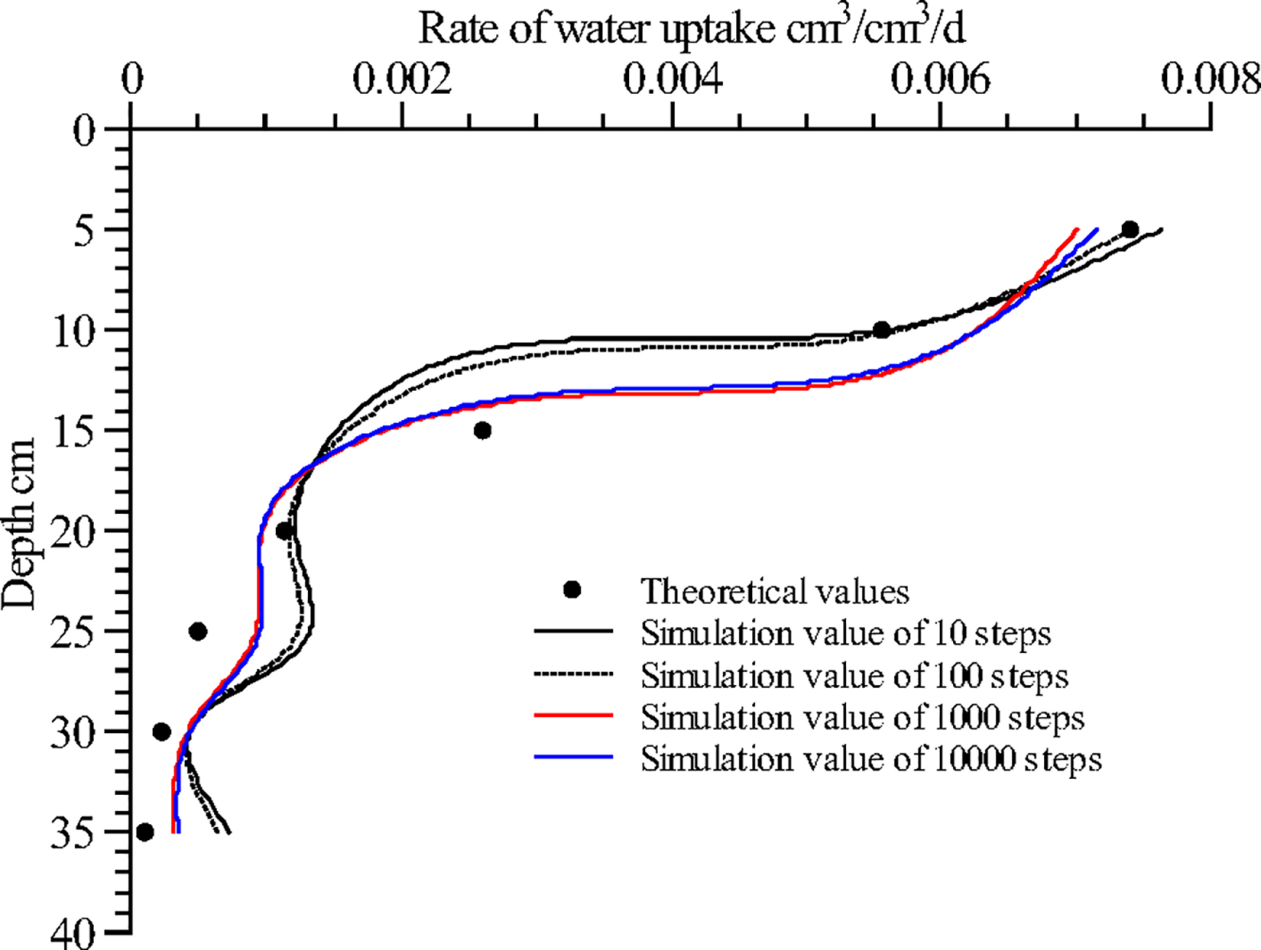
Calculated water uptake distribution in the soil profile at different time steps. Use inverse method to calculate distribution of water uptake in the soil profile at different time step (10, 100, 1000, 10000). Theoretical values are calculated from the root water uptake model developed by Shao AJ et al [33,34] (*S* = *ET*×*A*×[*e^-B*(*lnZ-C*)^2^]/Z).

Fig 2 illustrates the distribution of water uptake calculated at different time steps. The error analysis demonstrates that the accuracy was high at the time steps of 1000 and 10000; the corresponding RMSE, MAE, and ORE between the theoretical and calculated values fell between 4.51×10^−4^ and 4.59×10^−4^, between 7.12×10^−4^ and 7.50×10^−4^, and between 1.50% and 2.63%, respectively. When the time step was 10 or 100, the accuracy was relatively lower, with the corresponding RMSE, MAE, and ORE ranging from 5.16×10^−4^ to 5.76×10^−4^, from 9.88×10^−4^ to 1.07×10^−3^, and from 3.83% to 5.31%, respectively. However, the differences between these ranges and the ranges calculated at time steps of 1000 and 10000 were not significant. It was determined that the inverse method can yield satisfactory results with relatively small amounts of calculation at a time step of 100 or 1000.

### (2) Influence of hydraulic parameters on the results

**Fig 3:**
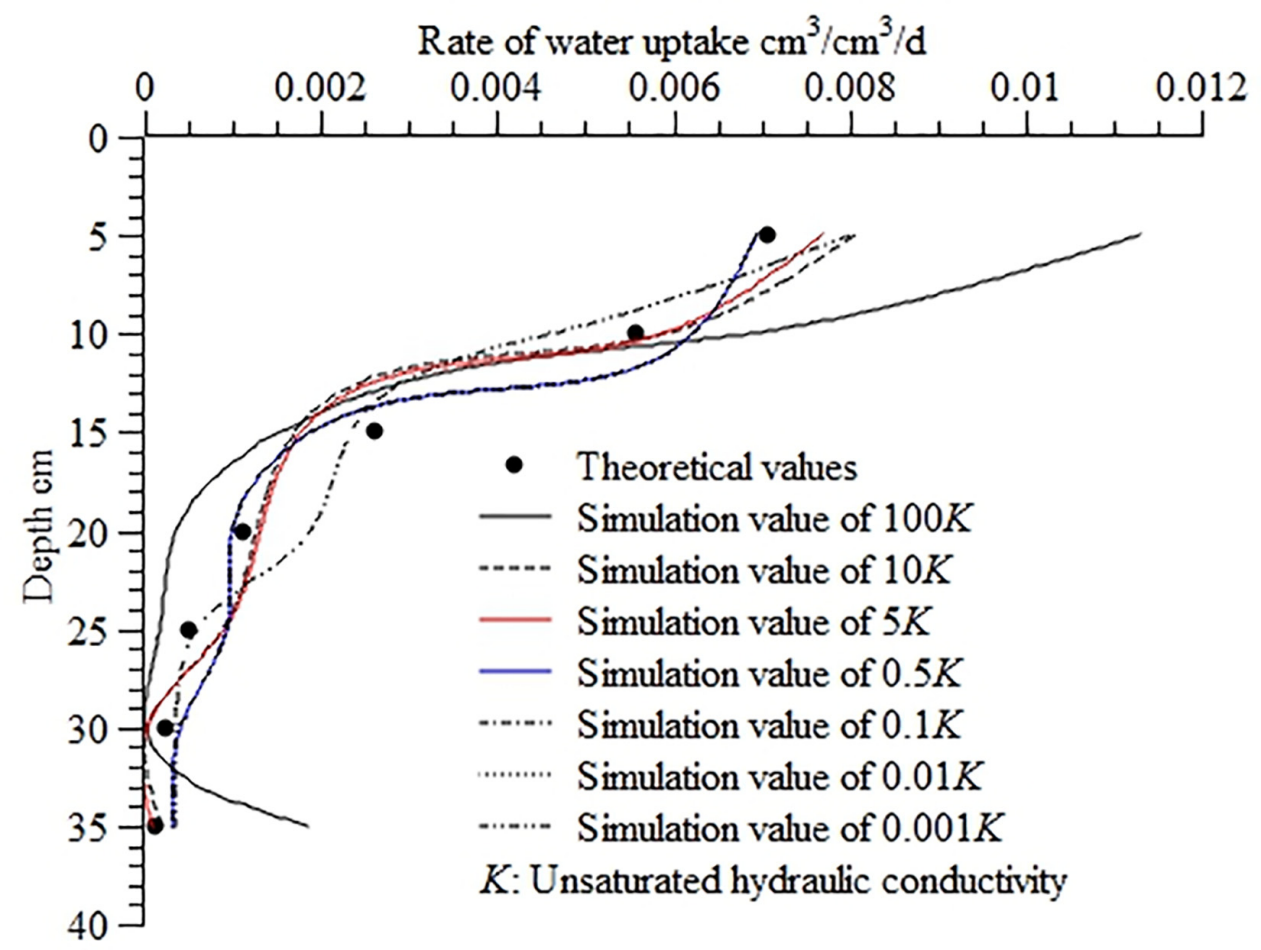
Calculated water uptake distribution in the soil profile at different levels of unsaturated hydraulic conductivity. Use inverse method to calculate distribution of water uptake in the soil profile at different unsaturated hydraulic conductivity (100*K*, 10*K*, 5*K*, 0.5*K*, 0.1*K*, 0.01*K*, 0.001*K*). 100*K* means the value of unsaturated hydraulic conductivity is increased by 100 times than the normal *K*, and 0.01*K* means the value of unsaturated hydraulic conductivity is reduced by 100 times than the normal *K*, Theoretical values are calculated from the root water uptake model developed by Shao AJ et al [33,34] (*S* = *ET*×*A*×[*e^-B*(*lnZ-C*)^2^]/Z).

Fig 3 shows the calculated water uptake distribution in the soil profile at different levels of unsaturated hydraulic conductivity. As shown in the figure, the correlation between the calculated values and theoretical values became weaker as the *K* value increased or decreased, indicating that changes in the value of *K* had an effect on the stability of the inverse method. Further, an increase in the *K* value exerts a greater effect on the results than a decrease in the *K* value. Overall, the errors didn’t vary significantly when the *K* value was between 0.001 *K* and 10 *K*, and the corresponding RMSE, MAE, and ORE fell between 4.42×10^−4^ and 5.97×10^−4^, between 7.05×10^−4^ and 9.95×10^−4^, and between 2.79% and 5.81%, respectively. At 100 *K*, the errors between the calculated and theoretical values were relatively large: the *RMSE* was 1.87×10^−3^, the MAE was 4.24×10^−3^, and the ORE was 28.84%.

**Fig 4:**
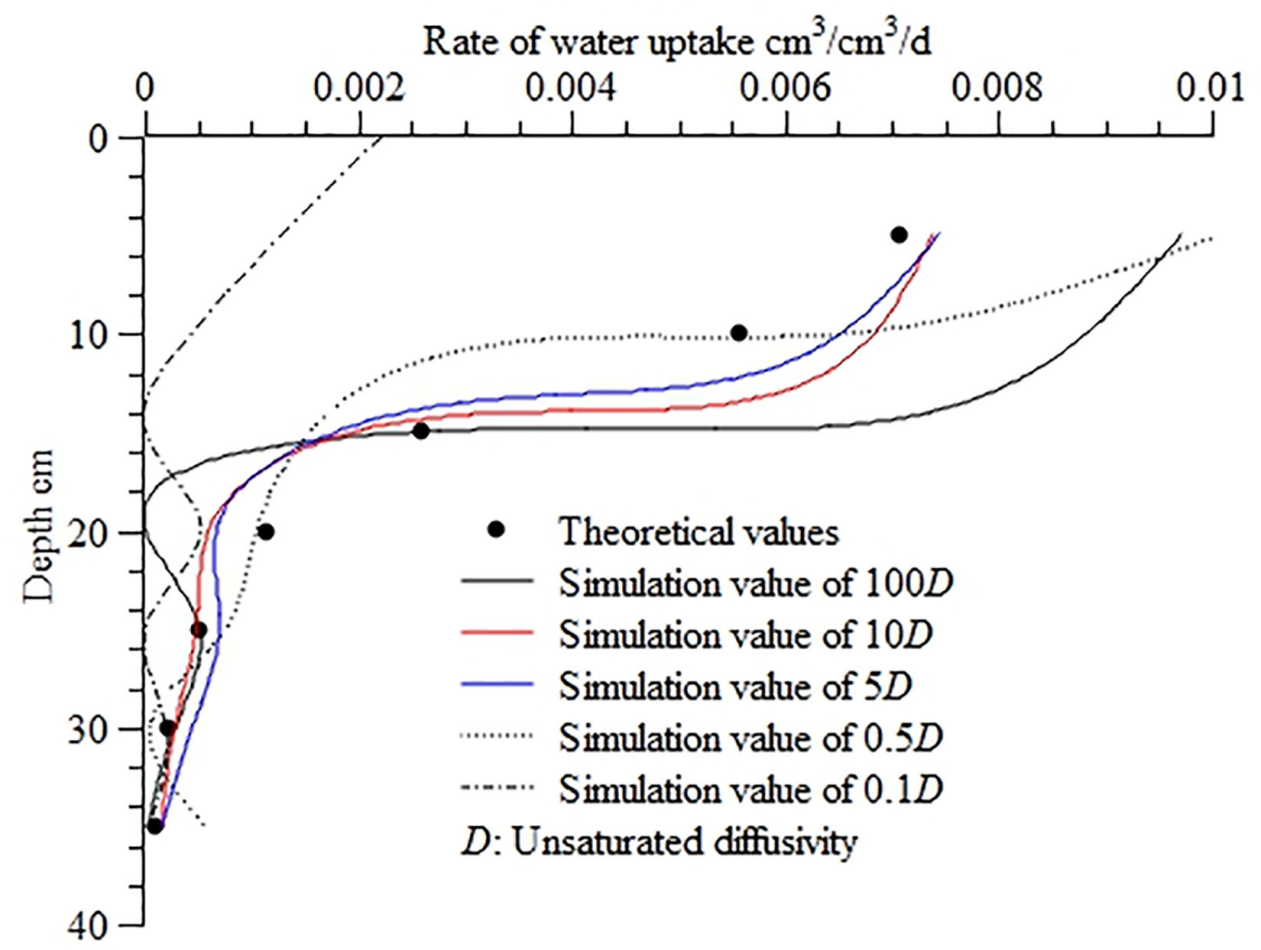
Calculated water uptake distribution in the soil profile at different levels of unsaturated diffusivity. Use inverse method to calculate distribution of water uptake in the soil profile at different unsaturated diffusivity (100*D*, 10*D*, 5*D*, 0.5*D*, 0.1*D)*, 100*D* means the value of unsaturated diffusivity is increased by 100 times than the normal *D*, and 0.01*D* means the value of unsaturated diffusivity is reduced by 100 times than the normal *D*, Theoretical values are calculated from the root water uptake model developed by Shao AJ et al [33,34] (*S* = *ET*×*A*×[*e^-B*(*lnZ-C*)^2^]/Z).

Fig 4 illustrates the calculated water uptake distribution in the soil profile at different levels of unsaturated diffusivity. The figure demonstrates that as the value of *D* increased or decreased the correlation between the calculated and theoretical values increased and then decreased, which is similar to the pattern observed at different levels of unsaturated hydraulic conductivity. However, variations in *D* had a smaller influence, comparatively. As the unsaturated diffusivity increased from 5 *D* to 10 *D*, the corresponding RMSE, MAE, and ORE ranged from 5.35×10^−4^ to 5.84×10^−4^, from 9.57×10^−4^ to 1.27×10^−3^, and from 3.01% to 3.17%, respectively. The unsaturated diffusivity of 100 *D* resulted in an RMSE of 1.64×10^−3^, MAE of 3.26×10^−3^, and ORE of 27.75%, indicating a relatively large influence on the calculation results. However, notable differences were observed between the effects of changes in the two hydraulic parameters. The results of the error analysis suggested that a decrease in *D* could significantly affect the stability of the inverse method. As the D value decreased from 0.5 *D* to 0.1 *D*, the RMSE, MAE, and ORE reached very high levels, at 1.28×10^−3^–1.14×10^−2^, 3.00×10^−3^–2.95×10^−2^, and 19.95–120.11%, respectively. Additionally, the inverse method was unable to give a solution at 0.01 *D*. These findings demonstrate that variations in unsaturated diffusivity, especially a decrease in unsaturated diffusivity, had significantly greater effects on the inverse method’s stability than variations in unsaturated hydraulic conductivity.

### (3) Effects of test error and instrument precision

**Fig 5:**
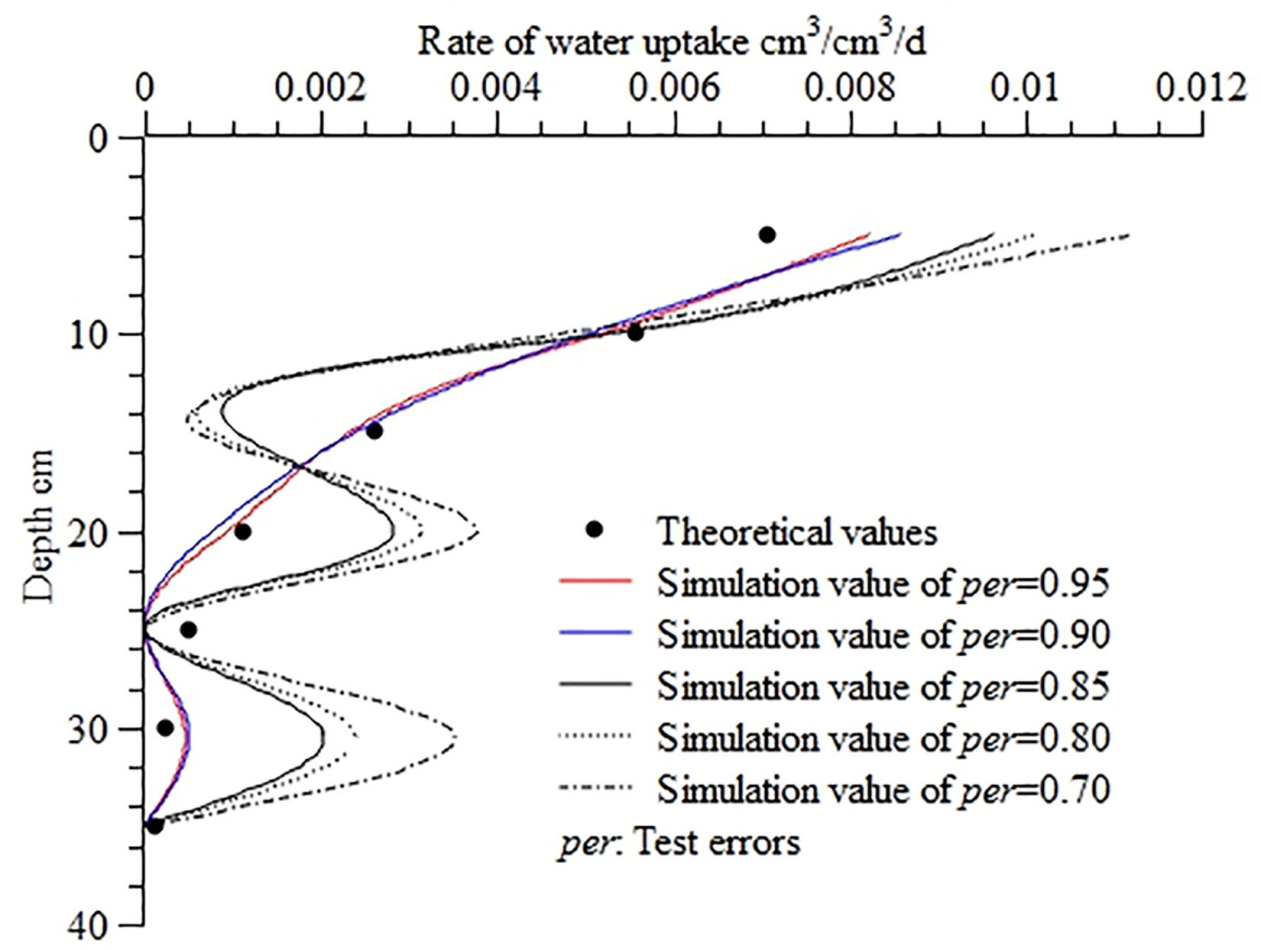
Calculated water uptake distribution in the soil profile at different test errors. Use inverse method to calculate distribution of water uptake in the soil profile at different test errors (*per* = 0.95, *per* = 0.90, *per* = 0.85, *per* = 0.80, *per* = 0.70), The bigger the value of *per*, the smaller the test error. Theoretical values are calculated from the root water uptake model developed by Shao AJ et al[33,34] (*S* = *ET*×*A*×[*e^-B*(*lnZ-C*)^2^]/Z).

Fig 5 shows the calculated water uptake distribution at different test errors. As the variation patterns show, the deviations of the calculated values from the theoretical values increased as the test error increased; when *per* ≥ 0.9, the RMSE, MAE, and ORE were between 5.15×10^−4^ and 6.51×10^−4^, between 1.15×10^−3^ and 1.49×10^−3^, and between 0.28% and 0.55%, respectively, ensuring relatively high accuracy. When 0.7 ≤ *per* ≤ 0.85, the deviations of the calculated values from the theoretical values were relatively significant: the RMSE, MAE, and ORE were high at 1.48×10^−3^–2.37×10^−3^, 2.56×10^−3^–4.12×10^−3^, and 21.04%-38.03%.

**Fig 6:**
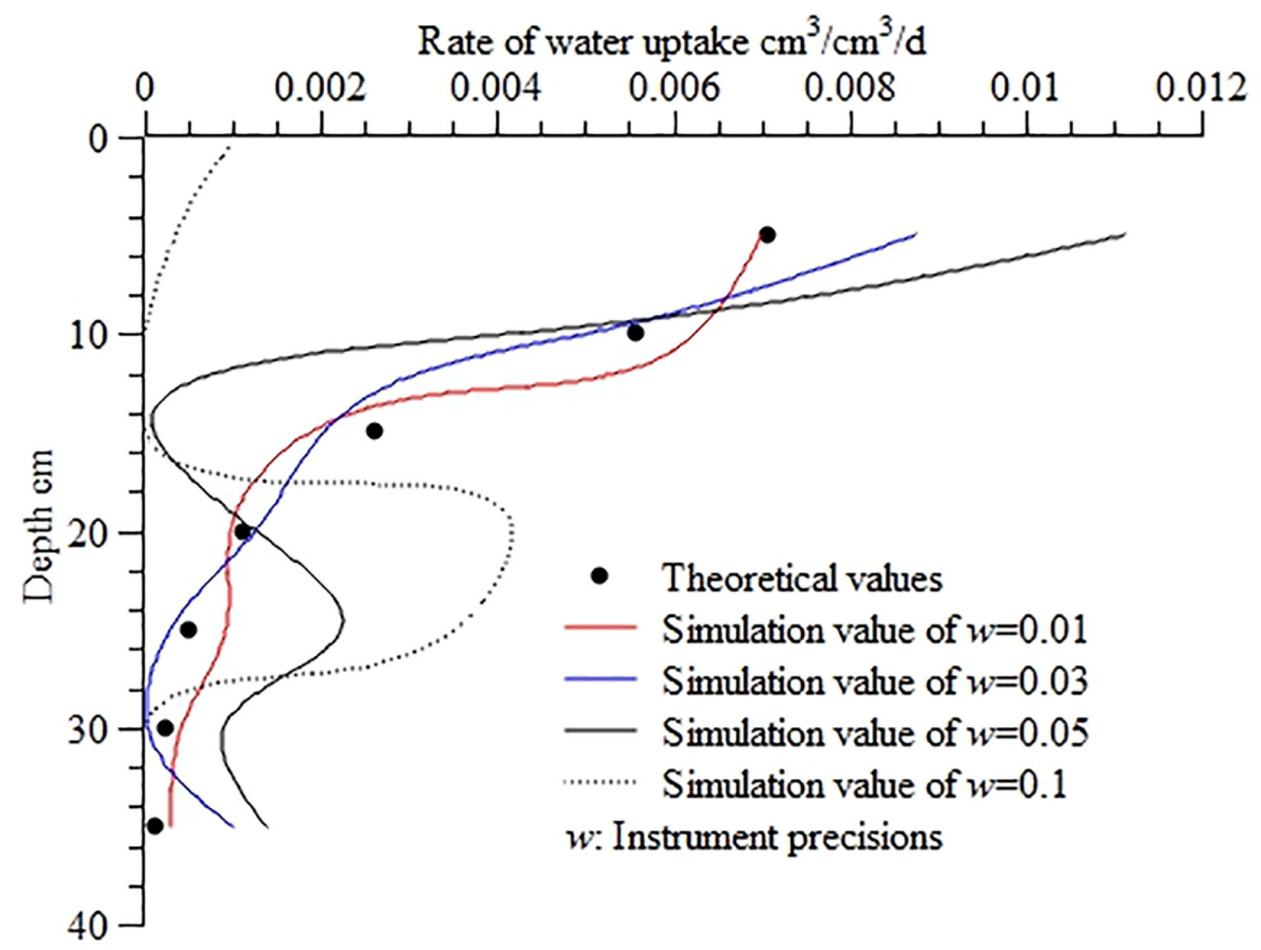
Calculated water uptake distribution in the soil profile at different instrument precisions. Use inverse method to calculate distribution of water uptake in the soil profile at different instrument precisions (*w* = 0.01, *w* = 0.03, *w* = 0.05, *w* = 0.1), The bigger the value of *w*, the smaller the instrument precisions. Theoretical values are calculated from the root water uptake model developed by Shao AJ et al[33,34] (*S* = *ET*×*A*×[*e^-B*(*lnZ-C*)^2^]/Z).

Fig 6 shows the calculated water uptake distribution at different levels of instrument precision. It was found that the deviations of the calculated values from the theoretical values increased with decreasing instrument precision; when *w* ≤ 0.03, the RMSE, MAE, and ORE fell within the ranges of 4.27×10^−4^–7.84×10^−4^, 7.12×10^−4^–1.68×10^−3^, and 3.41%-7.14%, respectively, ensuring relatively high accuracy of calculation. When 0.05 ≤ *w* ≤ 0.1, the RMSE, MAE, and ORE were high at 2.06×10^−3^–4.76×10^−3^, 4.05×10^−3^–1.01×10^−2^, and 23.13–44.66%, respectively.

### (4) Effect of layered soil and boundary conditions

**Fig 7:**
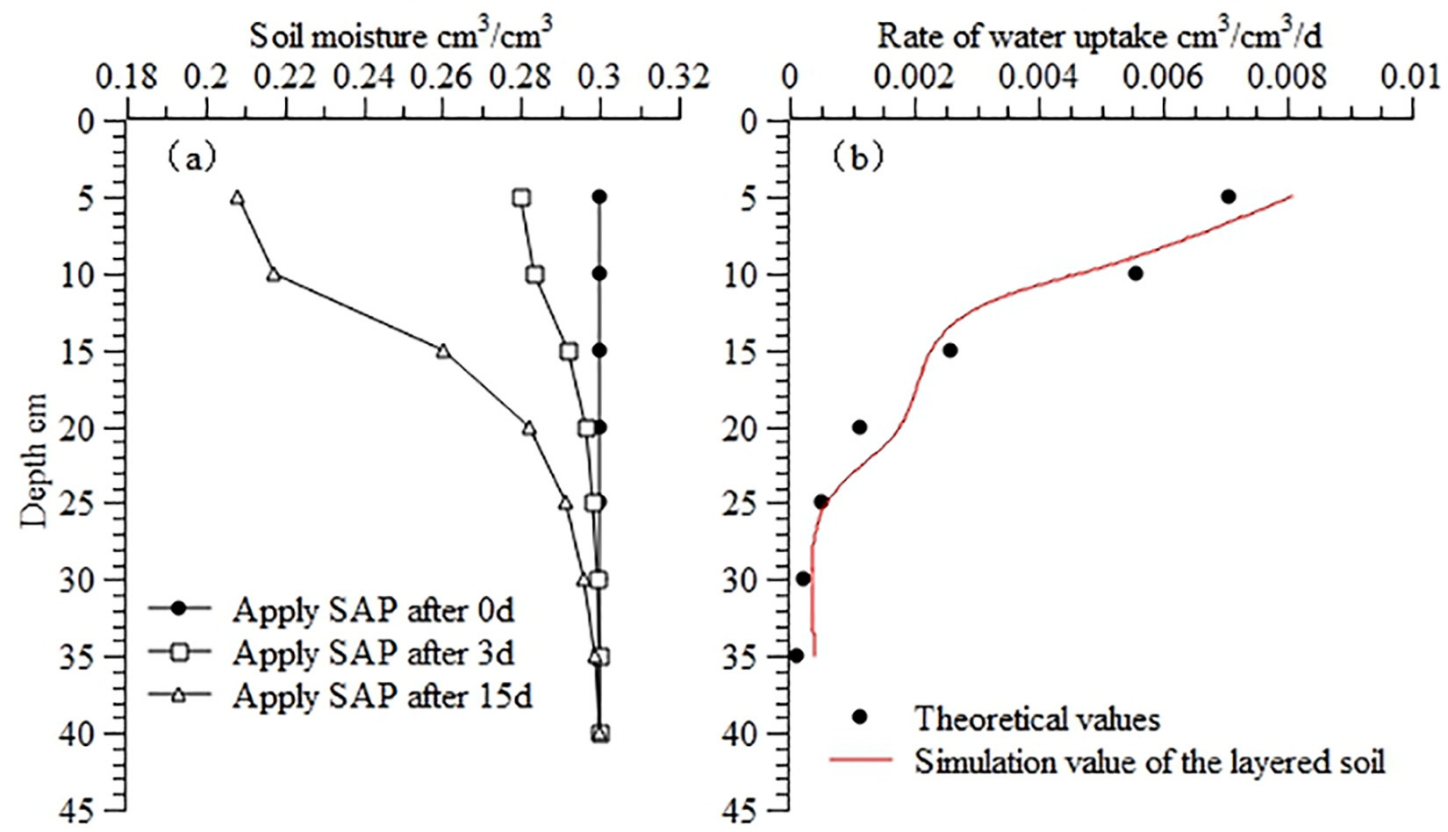
Theoretical moisture and calculated water uptake distribution in the profile of layered soil. (a) Theoretical moisture distribution; (b) Calculated water uptake distribution. Use inverse method to calculate distribution of water uptake in the profile of layered soil (sandy loam+silty soil), Theoretical distribution of moisture and water uptake are calculated from the root water uptake model developed by Shao AJ et al [33,34] (*S* = *ET*×*A*×[*e^-B*(*lnZ-C*)^2^]/Z).

Fig 7 illustrates the theoretical moisture in layered soil consisting of sandy loam and silty soil and the corresponding calculated water uptake distribution in the soil profile. The figure demonstrates relatively small differences between the calculated and theoretical values, with a RMSE of 5.86×10^−4^, a MAE of 1.02×10^−3^ and an ORE of 5.92%.

**Fig 8:**
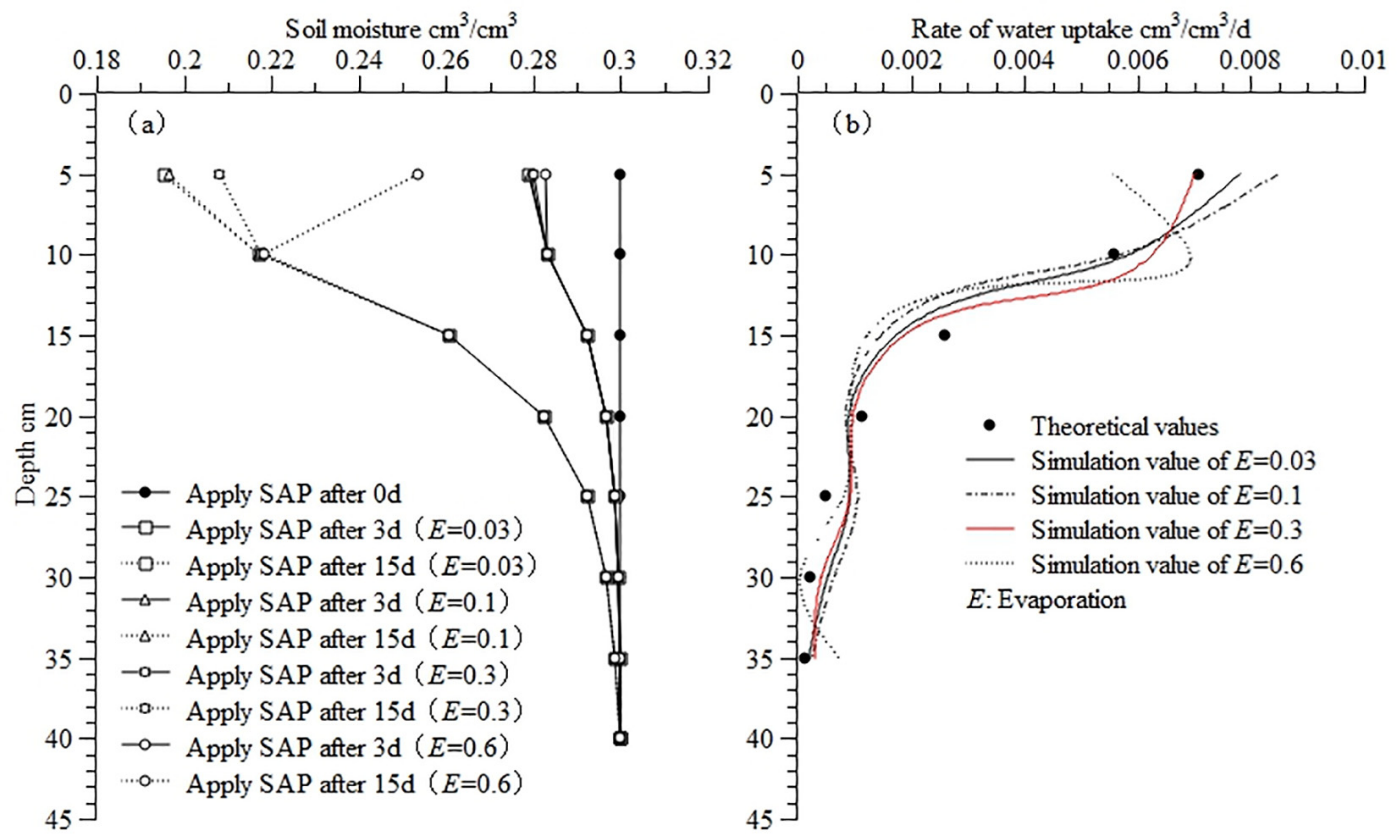
Theoretical moisture and calculated water uptake distribution in the soil profile at different rates of soil surface evaporation (a) Theoretical moisture distribution (b) Calculated water uptake distribution. Use inverse method to calculate distribution of water uptake in the soil profile at different rates of soil surface evaporation (*E* = 0.03, *E* = 0.1, *E* = 0.3, *E* = 0.6), The “*E*” stands for evaporation. All the distribution of water uptake in the soil profile are calculated from applying SAP 3d to 15d. Theoretical distribution of moisture and water uptake are calculated from the root water uptake model developed by Shao AJ et al[33,34] (*S* = *ET*×*A*×[*e^-B*(*lnZ-C*)^2^]/Z).

Fig 8 illustrates the theoretical moisture distribution and calculated water uptake distribution in the soil profile at different rates of soil surface evaporation. It was found that a higher rate of soil surface evaporation caused greater differences between the calculated and theoretical values. Error analysis suggested that as *E* increased, the errors increased, decreased, and increased again (Table 2), in a relatively moderate manner, with the RMSE, MAE, and ORE falling within the ranges of 4.27×10^−4^ to 9.68×10^−4^, 8.52×10^−4^ to 1.50×10^−3^, and 3.45% to 7.33%, respectively.

## Discussion

### Model of soil water movement under the effect of SAP

Few studies have been conducted to model soil water movement under the effect of SAP. Han YG et al[35] have investigated the patterns of dynamic variation in soil’s hydraulic parameters under the effect of SAP and constructed a model of water movement in bare soil. In this model, the duration of SAP’s effect on the soil’s hydraulic parameters, *T*, is a parameter independent of the duration of water movement time *t*, with *D(θ*,*T)* and *K(θ*,*T)* denoting the two hydraulic parameters. They solved the s equation to obtain an analytical solution based on assumptions and simplifications (which were achieved by replacing *D(θ*,*T)* in the Richards equation with average diffusivity *D* as an approximation). This analytical solution was shown to be capable of reflecting the dynamic effect of SAP on soil water movement. However, as this model mainly applies to bare soils, it is extremely difficult to analytically solve when root water uptake is considered, inhibiting further development of the model. The model of soil water movement under the effect of SAP described in this paper was also constructed based on variation in hydraulic parameters, but the model used in this paper regards the duration of SAP’s effect on the soil’s hydraulic parameters and the duration of water movement time as the same parameter *t*, with the two hydraulic parameters denoted by *D(θ*,*t)* and *K(θ*,*t)*. This model was solved by differentiation to obtain numerical solutions. The use of mesh generation in the differentiation method avoids the use of average values of hydraulic parameters when obtaining numerical solutions. In theory, this model is able to reflect, relatively well, the time-varying effects of SAP on the hydraulic parameters, it can also indicate the effect of soil layering caused by SAP. Further, the modeling and numerical method proposed in this paper provide a feasible approach to solving the source/sink term of root water uptake in the Richards equation as well as a basis for constructing models of soil water movement under the effect of SAP that consider water uptake by plant roots.

### The Inverse Method of Estimating Root Water Uptake and Its Stability

Since the rate of water uptake is not measurable, inverse methods for calculating it are important. The theoretical water uptake models in the inverse method of calculating the source/sink term of root water uptake referred to in this paper were constructed based on root length density[11,12]. However, considering the potential effect of SAP’s special functional property on plant roots, it is impossible to determine whether the rate of root water uptake is directly correlated with root length density in the presence of SAP. Therefore, use of the theoretical water uptake models constructed based on root length density would have an influence on the accuracy of calculated values obtained with the inverse method. In fact, it is unreasonable to use theoretical water uptake functions that were established based on root development indicators when the SAP’s effect on roots remains unclear. For this reason, this paper uses a theoretical water uptake model that was constructed based on the relationship between transpiration and root water uptake. The depth of the bottom of the zone that supplies water to plant roots is the only parameter in this model that is correlated with roots, and the correlation between them is weak. The use of the model in this study can minimize the potential effect of root development, thus ensuring the objectiveness of this study and accuracy of the results.

All the factors analyzed in the paper were shown to have an effect on the stability of the inverse method. Test error and instrument precision were found to have greater effects on the stability of the results of calculation than other factors, therefore, special attention should be paid to them. Significant test errors can greatly impair the accuracy of the calculated moisture distribution, which can in turn cause calculation errors due to the high dependence of the method on the accuracy of the calculated moisture distribution. Since instrument precision controls the goodness of fit of the moisture distribution, low instrument precision may obstruct the generation of smooth and accurate moisture distribution profiles that is required by the iteration method [36], and lead to calculation errors. Boundary conditions exerted a relatively small influence on the stability of the inverse method. The experimental results demonstrate that the proposed inverse method of calculating root water uptake applies to both homogeneous and layered soils. Normally, a soil’s hydraulic parameters are closely related to its texture; however, the soil’s texture will exert a slight influence on the results of the inverse iteration as long as the hydraulic parameter determination is sufficiently reliable. When Shi JC et al[8] applied inverse iteration to estimate nitrogen uptake by wheat roots they found that the layering of soil didn’t affect the results of the calculation. Additionally, intense evaporation from the soil surface was found to have an effect on the calculation results, as indicated by the errors in the calculated water uptake from the surface soil. Therefore, it is necessary to minimize soil surface evaporation during relative experiments in order to obtain more accurate measurements of root water uptake from surface soil. The analysis of the hydraulic parameters’ influence suggests that both unsaturated diffusivity *D* and unsaturated hydraulic conductivity *K* significantly influenced the stability of the inverse method, and variations in *D*, especially decreases, can exert much greater influence than variations in *K*. The results of error analysis showed that a decrease from 0.5 *D* to 0.1 *D* in unsaturated diffusivity resulted in great errors in the calculated values, and no result was yielded at 0.01 *D*. Previous research suggested that the application of SAP resulted in a significant decrease, at 0.24–0.30 *D*, in the soil’s unsaturated diffusivity of the treatment groups as compared to the control groups. Thus, it is reasonable to infer that when using the inverse method to calculate root water uptake under the effect of SAP, the calculation results will have high errors if the dynamic variation in *D* over time is not considered.

## Conclusions

1. The unsaturated water diffusivity and unsaturated hydraulic conductivity of soil under the effect of SAP were expressed as *D(θ*,*t)* and *K(θ*,*t)*, respectively. The residence time of SAP in the soil and the duration of the experiment were considered as the same parameter *t*, simplifying the previously proposed model in which the residence time of SAP in the soil and the duration of the experiment were two independent parameters. This simplification enables further development of this model. The modeling method and numerical method proposed here provide a basis for constructing models of soil water movement under the effect of SAP that consider water uptake by plant roots.
2. An inverse method was proposed for estimating the source/sink term of root water uptake in the model of soil water movement under the effect of SAP. Numerical testing was conducted to test this method. The test results suggest that time interval, hydraulic parameters, test error, and instrument precision significantly affected the stability of this inverse method, while time step, layering of soil, and boundary conditions exerted relatively smaller effects. A comprehensive analysis of the method’s stability, amount of calculation, and accuracy suggests that the proposed inverse method applies if the following conditions are satisfied: the time interval is between 5 d and 17 d; the time step is between 1000 and 10000; the test error is ≥ 0.9; instrument precision is ≤ 0.03; and the rate of soil surface evaporation is ≤ 0.6 mm/d.

## Supporting Information

S1 Appendix Data for Figs 1–8. (XLSX)
